# The effectiveness of the intervention model based on cognitive behavioral therapy in reducing risky sexual behaviors among MSM

**DOI:** 10.3389/fpubh.2026.1870879

**Published:** 2026-09-03

**Authors:** Xiaochen Xiang, Xiaobing Zhai, Yumeng Lei, Jialu Feng, Qiang Wang

**Affiliations:** 1 Hubei Province Key Laboratory of Occupational Hazard Identification and Control, Medical School, Institute of Infection, Immunology and Tumor Microenvironment, Wuhan University of Science and Technology, Wuhan, China; 2 Faculty of Applied Sciences, Macao Polytechnic University, Macau, Macao SAR, China

**Keywords:** cognitive-behavioral therapy, high-risk sexual behavior, microbiota, MSM, sexually transmitted diseases, unprotected anal intercourse

## Abstract

**Methods:**

Based on CBT theory and the Internet, we established an intervention model targeting high-risk sexual behavior patterns in MSM. We then conducted a randomized controlled trial (*n* = 50) to assess the impact of the CBT-based intervention on high-risk sexual behaviors and gut microbiota changes in this population.

**Results:**

Prior to the implementation of the intervention, 44% of the participants engaged in high-risk behaviors despite having better cognitive levels. However, preliminary findings from post-intervention investigations indicate that intervention measures based on CBT demonstrate greater efficacy compared to traditional methods. After intervention with CBT, the incidence of unprotected anal intercourse in the intervention group was significantly reduced compared to the control group (*p* = 0.016). Some bacterial taxa (e.g., *Bifidobacterium longum* and Prevotella) showed potential associations with behavioral changes following CBT; however, these microbiome-related findings are exploratory and hypothesis-generating, not conclusive.

**Conclusion:**

The high-risk sexual behaviors of MSM increase health risks. However, the method of intervention through the CBT courses can effectively correct the deviation between the cognition and practice. In this process, changes in gut microbiota were observed to be potentially associated with behavioral changes, but these associations are preliminary and warrant further investigation in confirmatory studies.

## Graphical abstract

**Figure:**
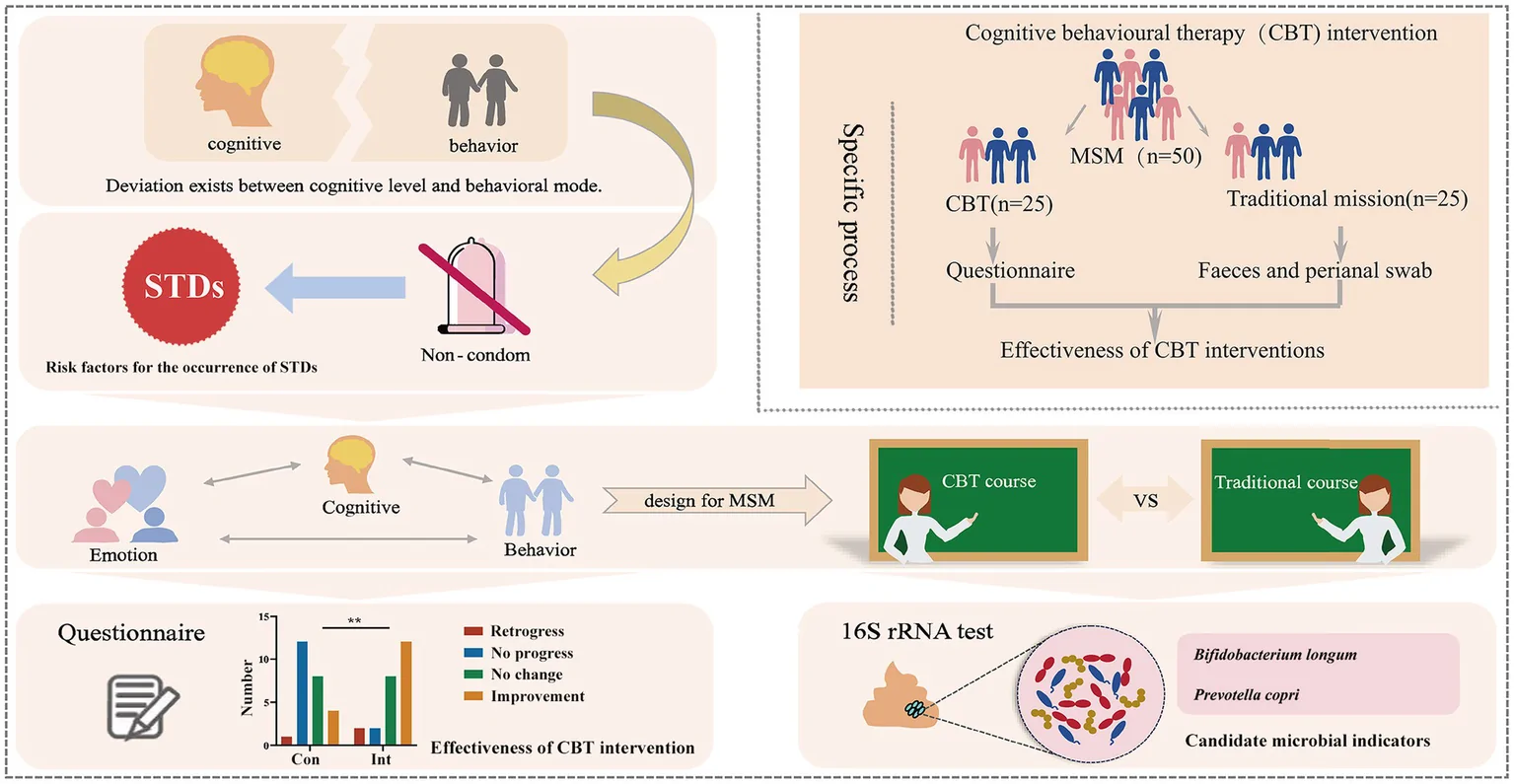

## 1 Introduction

High-risk sexual behaviors among men who have sex with men (MSM) represent a major global public health issue, exerting extensive impacts on the transmission of diverse Sexually Transmitted Diseases (STDs) (1). The types of STDs include AIDS, syphilis, and gonorrhea. Other STDs that are prioritized for prevention and control also include genital chlamydia infection, genital herpes, and genital warts. As per the data from the World Health Organization (WHO), approximately 20% of the sexually active population globally has been infected with at least one type of STD, and the incidence of STDs has been witnessing an upward trend in recent years (2). Furthermore, studies have shown that among MSM (3, 4), HIV infection and other STDs promote each other. Infection with syphilis and gonorrhea in MSM increases the risk of HIV infection. In China, the co-infection rate of HIV with STDs is on an annual upward trend. To this end, a more comprehensive understanding of the determinants of risk behaviors among MSM is requisite, and the implementation of effective public health interventions specifically tailored to this population is an urgent imperative (5).

Studying the behavioral characteristics of MSM populations and conducting targeted interventions for high-risk behaviors is a necessary approach to curb and reduce the transmission of STDs. Research results indicate that among MSM populations, 41.8% have multiple sexual partners, 17.4% occasionally use condoms, and 28.0% engage in receptive anal intercourse. The infection rates for HIV, *Treponema pallidum* (syphilis), and *Neisseria gonorrhoeae* are 5.7, 7.5, and 5.9%, respectively (6–8). Traditional intervention models for STDs typically involve health education on scientific knowledge within small population groups over a fixed period. While such intervention models can indeed reduce the incidence of STDs, but it still have limitations in effectiveness. This highlights the necessity of conducting in-depth research into the mechanism of STDs occurrence within MSM populations. A recent study found that among college students, their level of sexual health knowledge gradually increased during the first 2 years after enrollment, while the probability of engaging in risky sexual behavior also rose simultaneously (9). This indicates that the improvement in an individual’s sexual health knowledge is not completely consistent with their sexual behavior patterns. Such inconsistency may represent a major obstacle for traditional sexual health intervention models in preventing the occurrence of STDs. This phenomenon of the mismatch between sexual health knowledge and high-risk sexual behavior may also exist among MSM (10). Although they possess certain sexual health protection knowledge, they may still engage in high-risk sexual behavior decisions that contradict such knowledge due to factors such as beliefs and attitudes (11). In light of this, it is of paramount importance to introduce and apply cognitive-behavioral therapy (CBT). CBT is a set of treatment methods that aim to change maladaptive cognitions by altering thoughts, beliefs, and behaviors, thereby eliminating negative emotions and behaviors. During the treatment process, cognitive correction techniques and behavioral therapy techniques can be utilized, primarily encompassing two aspects: cognitive intervention and behavioral intervention (12–14). CBT is not a simple addition of cognitive theory and behavioral theory, but rather a development that addresses the shortcomings of each. It primarily focuses on the individual’s irrational cognitive issues, aiming to correct emotional and behavioral problems at their source by transforming the patient’s thought system. Currently, CBT has been widely applied in clinical settings. Case analyses show that the treatment approach combining CBT with clinical practice yields good results in the treatment of mental disorders such as depression and anxiety (15–17). CBT may be a potentially effective intervention for the phenomenon of cognitive and behavioral inconsistencies present in MSM. CBT-based interventions targeting high-risk sexual behavior among MSM are not simply knowledge–behavior correction. Instead, through classic techniques such as cognitive restructuring and behavioral skills training, focus on correcting erroneous beliefs, improving negative attitudes, and optimizing the decision-making process of high-risk sexual behavior, ultimately achieving effective intervention in such behaviors (18). This therapy can assist the MSM population in re-evaluating and adjusting their understanding and practice of safe sex, and is expected to become a key measure for reducing associated health risks within the MSM community.

The concealment of high-risk sexual behaviors among MSM presents a significant challenge in evaluating the effectiveness of behavioral interventions (19). Traditionally, evaluations of intervention efficacy targeting high-risk sexual behaviors among MSM have largely depended on questionnaire-based self-report surveys. Such scale-based assessments mainly capture behavioral information from a subjective perspective, highlighting the demand for multi-dimensional evaluation approaches to comprehensively reflect intervention effects. Expanding the methods for evaluating intervention effectiveness can help more accurately reflect the intervention outcomes. Moreover, among MSM engaging in high-risk behaviors, pathogens associated with STDs can be detected to varying degrees in the anal and rectal regions, the anus/rectum represents the most common site of infection (20–22). The microecological environment of the anal rectal region plays a crucial role in protecting the local area from infection by pathogenic microorganisms. High-risk sexual behaviors among MSM can directly disrupt this local microecological environment, and its imbalance increases the risk of pathogen infection. Studies have shown that pathogens associated with STDs, such as *T. pallidum*, *Trichomonas vaginalis*, *Haemophilus ducreyi*, *Chlamydia trachomatis* (lymphogranuloma venereum), *N. gonorrhoeae*, Mycoplasma, herpes simplex virus, and human papillomavirus, can be detected to varying degrees in the anal and rectal regions of MSM with high-risk behaviors. Infections with these pathogens enhance susceptibility to and transmission of HIV (23–25). The microbiome characteristics of the anal rectum in the MSM population may serve as potential biological evidence for evaluating the effectiveness of behavioral interventions. Therefore, we collected fecal and anal swab samples from MSM for the detection of gut microbiota and the microbiota ecology of the flora in the anal/rectal regions, respectively. Behavioral changes might induce detectable shifts in gut/perianal area microbiota ecology. We conducted an exploratory assessment of the microbiome as an objective indicator for a CBT intervention targeting high-risk sexual behaviors among MSM, providing preliminary experience and foundational data for the in-depth research on the intersection of prevention intervention studies and microbiology research.

To sum up, we conducted a sexual behavior disparity analysis within an exploratory pilot randomized controlled trial (RCT) in China, combined with microbiome profiling. The primary research outcome of this study is to explore whether the intervention under CBT courses can more effectively reduce the occurrence of high-risk sexual behaviors compared to traditional intervention models. The secondary research outcome is to explore the differences in microbial microecology among MSM under different intervention models. Through this combined population-based and microbiome analysis, our aim is to provide preliminary evidence for public health strategies aimed at alleviating the burden of sexually transmitted infections, thereby mitigating health risks associated with MSM.

## 2 Method

### 2.1 Design and determination of intervention effect evaluation tools

In this study, the “Health Literacy Questionnaire for the Prevention and Control of Sexually Transmitted Diseases and AIDS among MSM Population” was designed and developed through literature research, in-depth individual interviews, and the Delphi expert consultation method. The research team searched databases such as Web of Science, PubMed, and CNKI. Using subject terms or abstracts including “men who have sex with men (MSM),” “sexually transmitted diseases (STDs),” “acquired immunodeficiency syndrome (HIV/AIDS),” “intervention,” “preventive knowledge,” and “health literacy” for screening, a preliminary item pool for the “Health Literacy Questionnaire for the Prevention and Control of Sexually Transmitted Diseases and AIDS among MSM Population” was established. We selected 3–5 MSM individuals with different characteristics for in-depth interviews. We aimed to understand aspects such as the current situation of health education, psychological/behavioral characteristics, and willingness to receive STDs/AIDS prevention and treatment services among the interviewees. A total of 8 personnel from universities, medical institutions and other organizations were included in this study to form an expert consultation group. The expert authority coefficient (Cr) was used for expert selection, and the Cr coefficient was determined by three dimensions: the academic attainment level of the expert (q1), the basis for judgment (q2), and the familiarity with the research topic (q3). The calculation formula is as follows:


Cr=(q1+q2+q3)/3

The authority coefficient of the expert group = ΣCr/Number of experts.

(note: q1 represents the weight of academic level, q2 represents the weight of judgment basis, q3 represents the weight of familiarity level.)

In this research, we utilized the coefficient of variation (CV) as a metric to assess the degree of concordance among expert opinions. CV value less than 0.25 as an indication that the expert reviews of questionnaire items had reached a satisfactory level of consistency.

In summary, the selection criteria for each item of the questionnaire are as follows: (1) The average score of importance is ≥ 4 points; (2) The coefficient of variation is ≤ 0.25; (3) The selection rate is ≥ 80% and/or the full-score rate is ≥ 50%. For items that meet one or two of the above criteria, the research team discusses and revises them based on the experts’ suggestions. Subsequently, the revised items are included in the next round of expert inquiries. Items with an average importance score of less than 4 are deleted and will no longer be included in the next round of expert inquiries or the “Scale.” Detailed process of questionnaire development is provided in Supplemental file 2. This tool has limitations, which are manifested in that content validity was only verified through expert consultation.

### 2.2 Participant collection procedures in RCT studies

This randomized controlled trial focusing on STDs prevention among MSM was carried out in Wuhan, China. Participant recruitment began in May 2023. Formal intervention sessions were launched in July 2023 and lasted for 4 weeks. Fecal and anal swab samples of all participants were collected immediately upon completion of the intervention. No monetary compensation was provided to participants in this study. Normative implementation recommendations for CBT in group interventions, as proposed by relevant research and practical experience, suggest a group size of 25–45 participants. To ensure the high efficiency of personalized one-on-one interventions, this study adopted a smaller sample size of 25 per group, thereby recruiting a total of 50 participants across two groups. This RCT recruited 50 MSM participants (25 per group) for CBT intervention, following <Clinical Practice Manual for Group Cognitive-Behavioral Therapy of Anxiety Disorders> (9) recommending 25–45 per group. Inclusion: ≥18 years, self-identified MSM, consent provided. Exclusion: severe physical/mental illness affecting participation, recent (3-month) involvement in similar studies, or inability/unwillingness to consent. Recruitment of MSM participants was conducted using respondent-driven sampling (RDS). Three active MSM with varied demographic and behavioural characteristics were selected as initial seeds. Each seed was encouraged to recruit 3–5 eligible peer MSM. Newly recruited participants could further invite another 3–5 peers to participate, forming iterative recruitment waves. We planned to complete recruitment within three waves. After multiple rounds of peer referral, demographic and behavioural characteristics of participants were expected to stabilize, reducing sampling bias. This standardized controlled referral procedure differentiates formal RDS from conventional unregulated snowball sampling. The study subjects were sequentially numbered and allocated to groups corresponding to random numbers using a computer-assisted random number table method, with this randomization scheme generated via the website.^1^ Based on the data on questionnaire submissions and course viewings recorded in the software’s backend, subjects meeting the following criteria will be regarded as lost to follow-up or dropouts: (1) Course completion rate ≤ 75%; (2) Failing to submit questionnaires on time. The flowchart of recruitment and intervention is detailed in Supplementary material 1.

### 2.3 CBT course design and arrangement

This study was conducted by a professional project team consisting of 6 members, including 1 psychology professor, 2 medical professors, and 3 graduate students. The intervention team members possess expertise in mental health and preventive medicine, have long been engaged in research on HIV prevention interventions for young students, and have rich research and work experience in the prevention and control of AIDS and sexually transmitted diseases. In the design of our intervention, we followed the standardized implementation recommendations for CBT in group interventions as provided in the book (Clinical Practical Manual for Group Cognitive-Behavioral Treatment of Anxiety Disorders) published by Peking University Medical Press. We have established an instant messaging platform via WeChat, offering both real-name and anonymous, public and private interaction methods. CBT course was designed by researchers with experience in STDs/AIDS prevention and treatment as well as psychological and behavioral correction. The course consists of 8 class sessions, each lasting approximately 10–15 min. It is scheduled twice a week, and the intervention period is 4 weeks. The specific content is as follows: (1) Explanation of CBT Principles; (2) Basic Knowledge of STDs and Prevention/Treatment Matters; (3) Basic Knowledge of Other STDs and Prevention/Treatment Matters; (4) Conceptualization of Case-specific Issues; (5) Identification of Automatic Thoughts; (6) Identification of Intermediate Beliefs; (7) Identification and Correction of Core Beliefs; (8) Systematic Desensitization and Behavior Shaping Training. The detailed course objectives and course contents can be found in Supplementary file 2. The research team regularly pushed general prevention knowledge and information of STDs/AIDS to the enrolled subjects in the control group through the wechat interaction platform, and provided non-intervention course training and one-on-one consultation education. The subjects in the control group received regular evaluations, including the completion and submission of questionnaires. Both groups received the same 8 sessions with identical frequency to ensure consistency except for core intervention content. Based on the questionnaire submission and course viewing data recorded in the background, intervention subjects meeting the following criteria will be considered as lost to follow-up or dropouts: (1) Course completion rate ≤75% (2) Failure to submit the questionnaire on time. All data information is collected through anonymized online questionnaires, which effectively protects the privacy of participants.

### 2.4 Control condition

Participants in the control group received routine HIV/STI prevention education delivered in a didactic, lecture-style format, covering transmission routes, risk reduction, and regular testing recommendations, along with non-directive one-on-one supportive counseling (e.g., answering questions and providing reassurance) without any cognitive-behavioral techniques. Unlike the intervention group, control participants were not engaged in cognitive homework or behavioral practice. All control session scripts were reviewed weekly by the supervising psychologist to ensure the absence of CBT core components. This design ensured that any observed differences between groups could be attributed to the CBT-specific active ingredients rather than to differential attention, contact, or information exposure.

### 2.5 Sequencing experimental procedure

The microbiome’s DNA was extracted from fecal and anal swab samples using the FastDNA® Soil Spin Kit. The quality of the extracted DNA was detected by 1% agarose gel electrophoresis, and the DNA concentration and purity were measured using a NanoDrop2000 (Thermo Scientific, USA). Using the extracted DNA as a template, the full-length 16S rRNA gene was amplified by PCR with barcoded primers 27F (5′-AGRGTTYGATYMTGGCTCAG-3′) and 1492R (5′-RGYTACCTTGTTACGACTT-3′). Library preparation using the SMRTbell Prep Kit 3.0: (1) DNA damage repair; (2) end repair; (3) adapter ligation. Sequencing was performed using the PacBio Sequel IIe System (Shanghai Meiji Biomedical Technology Co., Ltd.). HiFi reads were generated from the sequenced subreads via the CCS mode of SMRT-Link v11.0 for subsequent data analysis. Separate data for each sample based on barcode sequences, perform length filtering and direction correction, and retain sequences ranging from 1,000 to 1800 base pairs.

### 2.6 Statistical analysis

Statistical analyses compared post-intervention sexual behavior patterns between groups using chi-square and nonparametric tests. All analyses used R 4.3.2 and GraphPad Prism 9.0, with statistical significance set at *p* < 0.05. Microbiome data analysis was conducted on the Majorbio Cloud Platform^2^ as follows: Alpha diversity indices such as Chao 1 and Shannon were calculated using the Mothur software^3^, and inter-group differences in alpha diversity were analyzed using the Wilcoxon rank-sum test. Principal Coordinates Analysis (PCoA), based on the Bray-Curtis distance algorithm, was employed to examine the similarity of microbial community structures between samples. Additionally, PERMANOVA non-parametric testing was used to analyze whether differences in microbial community structure between sample groups were significant. Statistical significance was defined as *α* = 0.05, and a *p*-value less than 0.05 was considered statistically significant.

## 3 Result

### 3.1 Within the MSM population, limited scientific understanding, along with the presence of inaccurate beliefs and risky behaviors, plays a significant role in increasing vulnerability to STDs

#### 3.1.1 Demographic characteristics

In our study, 50 subjects were recruited via peer-driven sampling and randomly assigned to a CBT intervention group or a control group. Subject ages ranged from 18 to 60 years. One subject in the intervention group dropped out midway through the study and was classified as lost to follow-up, while all other subjects completed all scheduled sessions. Comparison of demographic characteristics (e.g., age, residence, income, education) showed no significant differences between groups (*p* > 0.05), indicating comparability. See Table 1.

**Table 1:** Basic information of the research subjects.

Variate	Categorization	Int	Con	*X^2^*	*p*
Age	18–35	18	17	0.095	0.758^#^
	36–60	7	8		
Long-term place of residence	City	23	25		0.484^*^
	Village	1	0		
	Town	1	0		
Income	≤50,000	10	10	4.762	0.190
	50,000–100,000	8	13		
	100,000–200,000	6	1		
	≥200,000	1	1		
Status of romantic involvement	Still single	12	9	1.095	0.895
	Unmarried and single	3	3		
	Unmarried but not single	5	7		
	Divorced or widowed	1	2		
	Married	4	4		
Educational level	Junior high school and below	0	1		0.481^*^
	High school/technical secondary school	5	9		
	Undergraduate/Junior college	17	13		
	Master	3	2		
Occupation	Professional technicians	9	5	8.220	0.222
	Personnel in public institutions	0	4		
	Business personnel	2	4		
	Service industry workers	6	7		
	Agricultural workers	0	0		
	Industrial workers	1	1		
	Student	6	2		
	Unemployed individuals	1	2		
Sex-related education	Yes	0	1		1^*^
	No	25	24		
Homosexual sexual behavior	Yes	24	25		1^*^
	No	1	0		

^#^indicates that the expected count in a cell is greater than or equal to 1 but less than 5, so the continuity—corrected parameter is used; ^*^indicates that the expected count in a cell is less than 1, so the Fisher’s exact test is used.

#### 3.1.2 MSM generally demonstrate a good understanding of AIDS and sexually transmitted diseases

**Figure 1:**
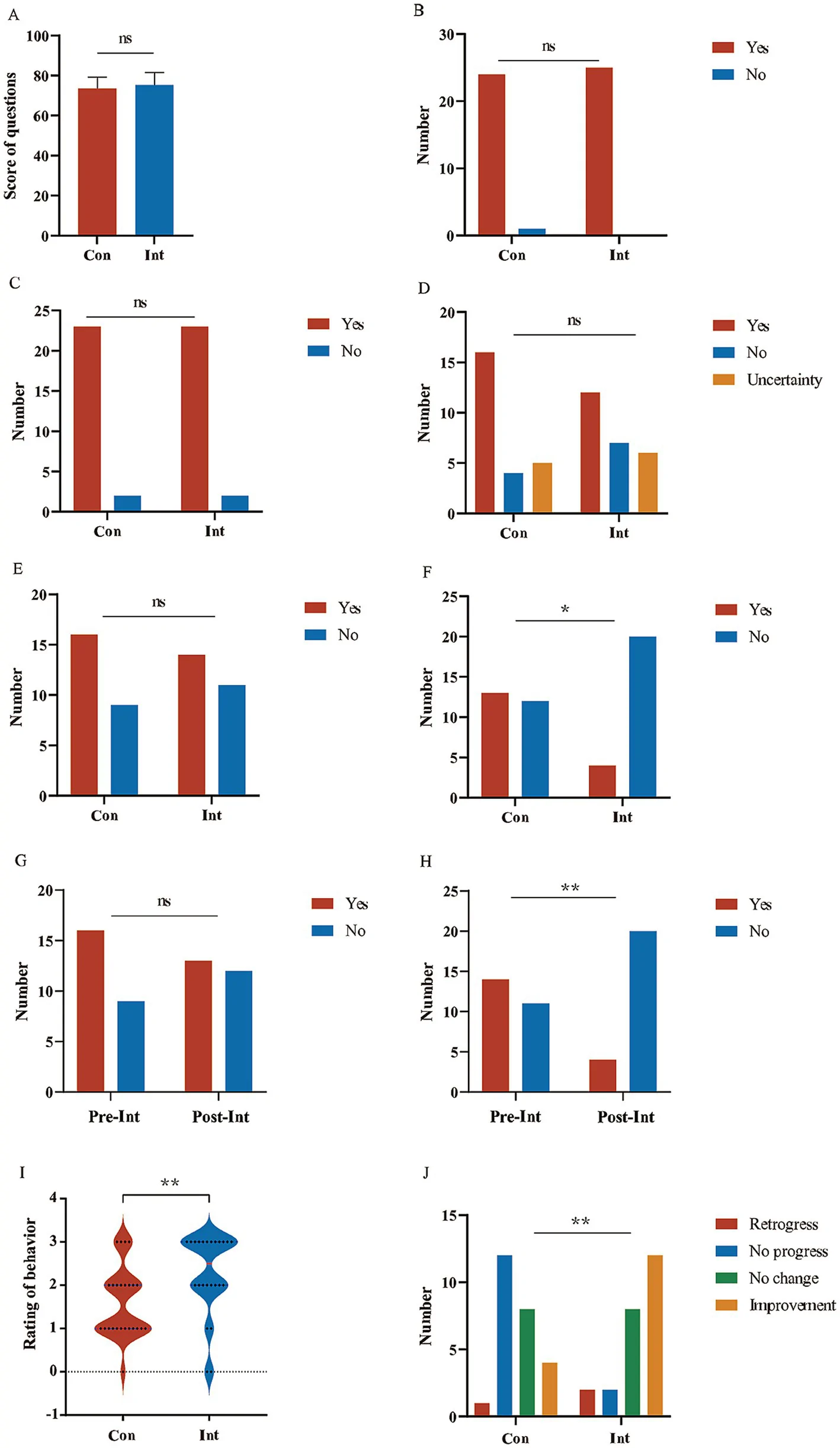
Alterations in the cognition, attitudes, and behaviors of MSM pre- and post-CBT intervention. **(A)** Scores of the two groups on the STDs knowledge questionnaire before the CBT intervention. **(B)** Willingness to use condoms (prior to intervention). **(C)** Willingness to pursue health screening following high-risk sexual activities (prior to intervention). **(D)** Whether condoms were utilized during the initial occurrence of homosexual sexual behavior. **(E)** Condomless anal sex occurred in the past month (prior to intervention). **(F)** Condomless anal sex occurred in the last month (subsequent to the intervention). **(G)** Condomless anal sex occurred in the past month (Con group). **(H)** Condomless anal sex occurred in the past month (Int group). **(I, J)** The frequency of condom use by participants during sexual activity in the recent month was categorized into four levels: Never, Occasionally, Frequently, and Every Time. Changes in participants’ related behaviors were assessed across four dimensions: Retrogress, No Progress, No Change, and Improvement, which were separately quantified as scores from 0 to 3. Violin plots were adopted for the statistical analysis of the above data. **(I)** The scores of behavioral patterns (subsequent to the intervention). **(J)** The categorization of behavioral patterns (subsequent to the intervention). An asterisk (^*^) denotes a statistically significant difference in this index between the groups. For the analysis of differences, non-parametric tests, chi-square test, were utilized. ^*^p < 0.05. ns, No significance; Con, Control group; Int, Intervention group.

Survey data from an MSM-focused STD/AIDS Health Literacy Questionnaire were analyzed for disparities between intervention (Int) and control (Con) groups. In the comparison of cognitive level scores between the two groups of participants prior to intervention implementation, the mean score for the intervention group was 75.25 (95% CI: 68.95, 81.55), and for the control group, it was 73.50 (95% CI: 68.01, 78.99). Both groups exhibited a relatively high level of knowledge regarding sexually transmitted diseases/HIV (Figure 1A), with no significant difference observed (*p* = 0.79). On the other hand, both groups of participants exhibited similarly high levels of positive attitudes (Figures 1B,C). Within the intervention group, all participants demonstrated a high willingness to use condoms, whereas only one participant in the control group lacked this willingness. The potential for improvement was limited in both groups, with no significant differences observed (*p* = 0.99). A similar phenomenon was observed regarding the willingness of both groups to actively seek medical testing following high-risk sexual behavior (*p* = 0.99).

Analysis of high-risk sexual behaviors related to MSM revealed that a considerable proportion of individuals in both groups reported not using or being uncertain about using condoms during their first sexual encounter (Figure 1D). Specifically, 12 individuals in the intervention group explicitly reported using condoms during their first sexual encounter, while 7 explicitly reported not using them; in the control group, 16 individuals explicitly reported using condoms during their first sexual encounter, and 4 explicitly reported not using them. There was no statistically significant difference between the two groups in this regard (*p* = 0.48). However, this result indicates the presence of notable cognitive-behavioral inconsistency within the MSM population.

#### 3.1.3 Alterations in sexual behavior patterns among MSM subsequent to CBT intervention

Unprotected anal intercourse is the primary high-risk sexual behavior among MSM, and thus serves as the main behavioral outcome observed in this study. The baseline survey showed that 9 subjects in the control group had recently engaged in unprotected anal intercourse, with an incidence rate of 36%, while 11 subjects in the intervention group had recently engaged in unprotected anal intercourse, with an incidence rate of 44%. There was no significant difference in the incidence of recent unprotected anal intercourse (UAI, unprotected anal sex that occurred within the past month) between the two groups (*p* > 0.05). Following a CBT-based curriculum intervention, a significant difference emerged (*p* = 0.016), with the intervention group having fewer individuals reporting UAI (Figures 1E,F). Within-group analysis found no change in UAI incidence over time for the control group (*p* > 0.05), but a significant decrease occurred in the intervention group post-CBT (*p* = 0.004; Figures 1G,H). Post-intervention, the intervention group had significantly higher behavioral grade scores than controls (*p* = 0.008; Figure 1I). Among those with prior UAI, the intervention group had more individuals categorized as “improvement” and fewer as “no improvement” compared to controls (*p* = 0.009; Figure 1J), This indicates that intervention models based on CBT course are associated with a reduction in high-risk behaviors.

### 3.2 The response of the gut/perianal microbiota ecology in the MSM population to the behavioral changes resulting from CBT intervention

#### 3.2.1 An overview of 16S rRNA gene sequencing data

**Figure 2:**
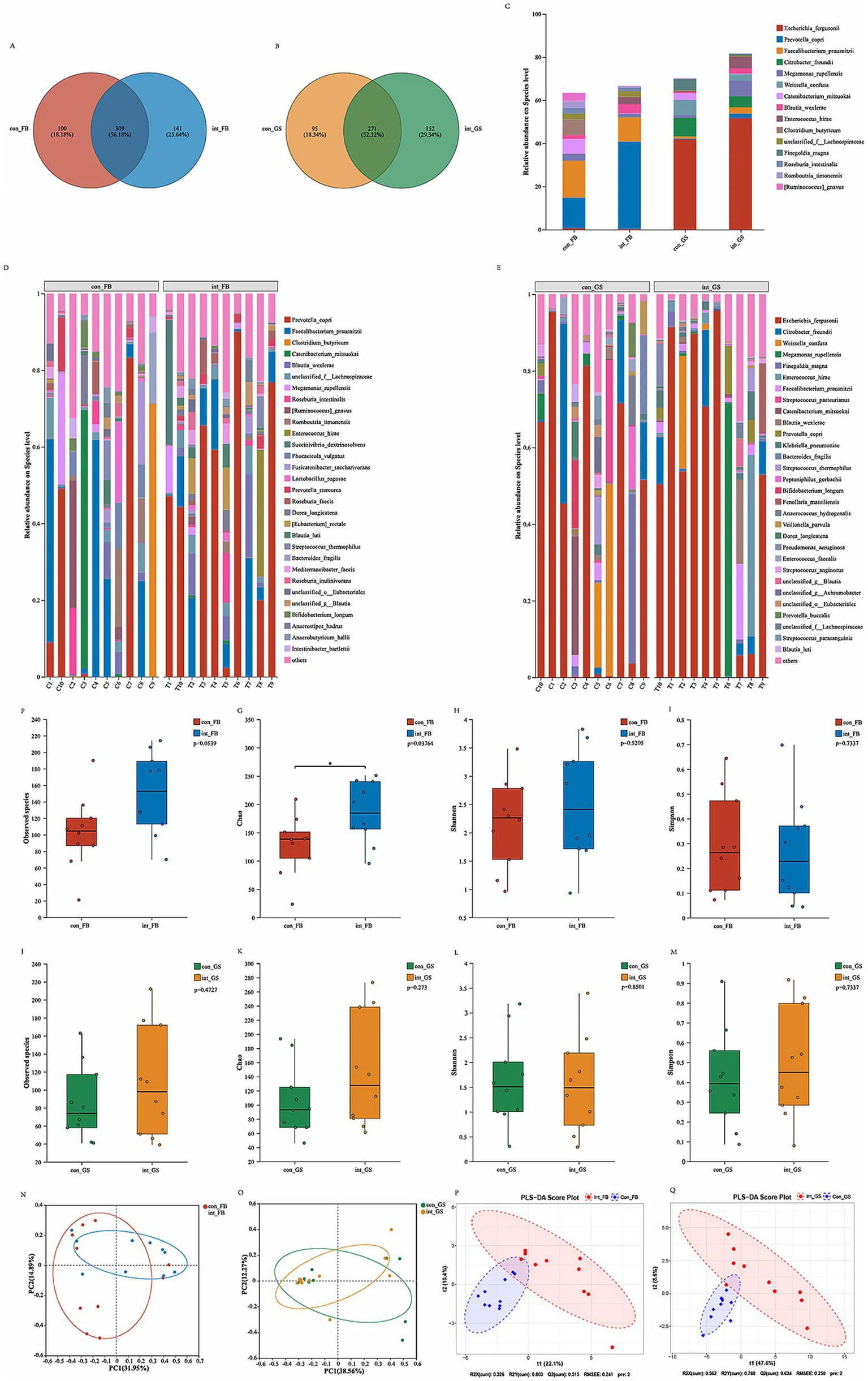
Taxonomic characteristics of gut and perianal microecology in the Int group and Con group. **(A, B)** OTUs Venn diagram analysis. **(C)** Stacked column charts of the percentage of colony composition at the species level. **(D)** Column chart of the colony structure at the species level of fecal samples. **(E)** Column chart of the colony structure at the species level of anal swab samples. **(F–M)** Comparison of alpha diversity. **(N)** PCoA analysis chart of fecal samples. **(O)** PCoA analysis chart of anal swab samples. **(P)** PLS-DA analysis diagram of fecal samples. **(Q)** PLS-DA analysis diagram of anal swab samples. FB, Fecal sample; GS, Anus swab sample; Con, Control group; Int, Intervention group.

Venn diagrams showed OTU counts in fecal and anal samples for Con and Int groups (Figures 2A,B). Fecal: Con had 409 OTUs, Int had 450, with 309 shared. Anal: Con had 366 OTUs, Int had 423, with 271 shared. Species source analysis (Supplementary Figure 1) revealed the Pan/Core curve plateaued, indicating sufficient sampling depth for sequencing. This provided a basis for comparing specific bacterial taxa differences between groups.

Fecal samples: Dominant species were *Prevotella copri* (27.39%), *Faecalibacterium prausnitzii* (14.12%), *Clostridium butyricum* (3.58%), *Catenibacterium mitsuokai* (3.49%), and *Blautia wexlerae* (3.09%) (Figure 2D). Int group had higher *P. copri* (40.56% vs. 14.22%) and *B. wexlerae* (4.29% vs. 1.88%) than Con group (Figure 2C).

Anal swabs: Dominant species were *Escherichia fergusonii* (46.69%), *Citrobacter freundii* (6.84%), *Weissella confusa* (5.17%), *Megamonas rupellensis* (4.20%), and *Finegoldia magna* (2.06%) (Figure 2E). Con group had higher *C. freundii* (8.62% vs. 5.05%) and *W. confusa* (7.22% vs. 3.11%).

#### 3.2.2 Comparison of the richness and diversity of gut and perianal microbiota ecology

We utilized dilution curves in conjunction with diversity indices to evaluate sequencing depth. Dilution curves and diversity indices (Shannon/Simpson) flattened, indicating sufficient sequencing depth and sample size (Supplementary Figure 2).

Alpha-diversity indices (Table 2 and Figures 2F–M) assessed microbial richness/diversity. For fecal samples, the Int group showed higher Observed-species (150.1 ± 49.05 vs. 103.1 ± 44.01, *p* = 0.054) and significantly higher Chao index (185.5 ± 53.97 vs. 128.86 ± 50.93, *p* = 0.038) than the Con group, suggesting greater gut microbiota richness in the intervention group.

**Table 2:** Comparison of alpha-diversity indices of gut and anal microflora.

Group		Observed-species	Chao1	Shannon	Simpson
Fecal	Con	103.1 ± 44.01	128.86 ± 50.93	2.17 ± 0.79	0.29 ± 0.20
	Int	150.1 ± 49.05	185.5 ± 53.97	2.50 ± 0.99	0.26 ± 0.21
	*p*	0.054	0.038^*^	0.52	0.73
Anal swab	Con	85.2 ± 41.01	105.43 ± 49.32	1.62 ± 0.90	0.42 ± 0.25
	Int	107.9 ± 60.68	146.12 ± 79.22	1.54 ± 0.97	0.49 ± 0.28
	*p*	0.47	0.27	0.85	0.73

Non-parametric tests were used to assess the statistical differences between the two groups of samples. The data are presented in the form of mean ± standard. ^*^indicates that there is a statistical difference in this parameter between the two groups, with ^*^p < 0.05.

Beta diversity assessed microbial community differences. PCoA of fecal samples (Figures 2N,O) showed clear separation between Con and Int groups (PC1 = 31.93%, PC2 = 14.89%), suggesting significant structural differences. Anal swab separation was less distinct. PLS-DA (Figures 2P,Q) confirmed distinct separation patterns for both sample types. Gut microbiota differences between groups were more pronounced than perianal microbiota differences.

#### 3.2.3 Differential analysis

**Figure 3:**
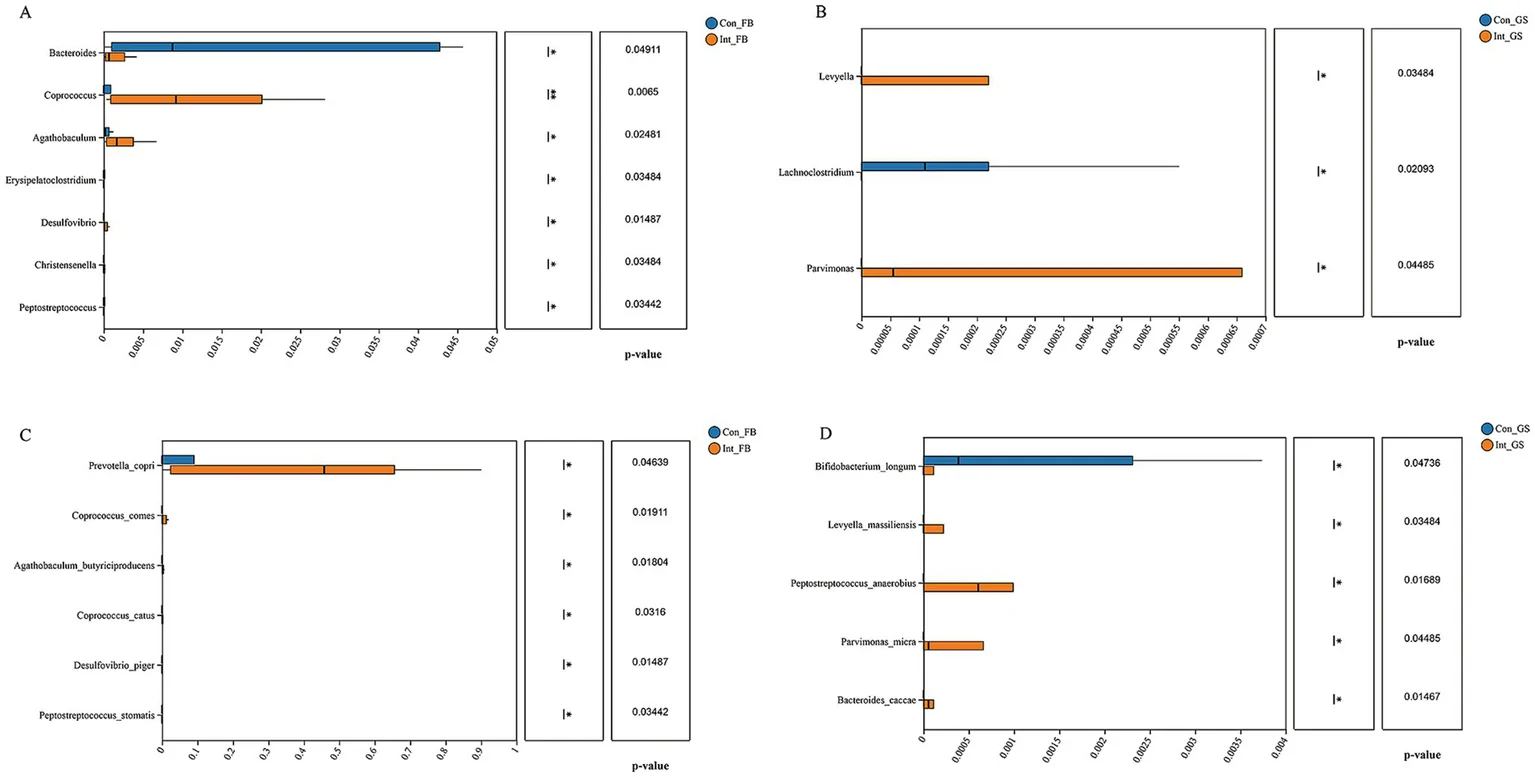
Analysis of the differences in the intestinal/perianal microecological structure between the Con group and the Int group. **(A)** Differential analysis of the bacterial communities in two groups of fecal samples at the genus level. **(B)** Differential analysis of microbial communities at the genus level between two groups of anal swab samples. **(C)** Analysis of microbial differences at the species level between two groups of fecal samples. **(D)** Analysis of microbial differences at the species level between two groups of anal swab samples. FB, Fecal sample; GS, Anus swab sample; Con, Control group; Int, Intervention group.

The Wilcoxon rank-sum test was used to analyze whether there were differences in the composition of intestinal or perianal microbiota between the intervention group and the control group. Given the exploratory nature of this pilot study and the hypothesis-generating objective, we did not adjust *p*-values for multiple comparisons. This conservative choice prioritizes the detection of potential microbial signals that may warrant further investigation, at the cost of an increased risk of false-positive findings. Therefore, the differentially abundant taxa identified below should be interpreted as candidate signals rather than confirmed differences, and all nominally significant associations require validation in independent, larger cohorts. At the genus level, the relative abundance of Bacteroides species in fecal samples of the intervention group was significantly lower than that of the control group, while the relative abundances of Coprococcus and Agathobaculum species were higher (Figure 3A); At the genus level, perianal swab samples showed that the intervention group had relatively higher species abundance of Levyella and Parvimonas compared to the control group, while Lachnoclostridium species abundance was relatively lower (Figure 3B). At the species level, the species abundance of *P. copri* strains in the fecal samples of the intervention group was significantly higher than that of the control group (Figure 3C). Significant differences in species abundance were observed between the two groups for strains such as Levyella massiliensis, *Peptostreptococcus anaerobius*, *Parvimonas micra*, *Bacteroides caccae*, and *Bifidobacterium longum* in the anal swab samples. Notably, the species abundance of *B. longum* was significantly higher in the control group (Figure 3D).

## 4 Discussion

STDs represent a significant global health challenge, affecting both developing and developed countries, with a particularly pronounced impact on young populations. Compared to other population groups, the MSM population faces a higher risk of infection or transmission of STDs, which is associated with high-risk factors such as having multiple sexual partners, engaging in unprotected sex, and bidirectional sexual behavior. Studies have shown that 53.0% of MSM engage in unprotected anal intercourse (26). The baseline survey data of MSM participants in this study showed a 40% rate of unprotected anal intercourse, which is close to the data reported in previous studies. We examined the research subjects’ situations regarding AIDS-related risk awareness, willingness to use condoms, learning enthusiasm, and self-acceptance of sexual orientation from the attitude dimension before and after the intervention. According to the research data, both groups of participants tended to choose positive answers; however, this contradicts the behavioral data. Chen Lingxue’s team also observed a similar phenomenon. In their 2-year natural follow-up study of college students, they found that most students held positive attitudes toward sexual behavior; however, there was a certain proportion of students engaging in high-risk sexual behaviors during the second academic year (9). A cross-sectional survey by Wang Honghong’s team also found that most students are aware that using condoms can reduce the risk of HIV infection, but condom use during sexual activity remains low (27). This suggests that traditional intervention models for STDs may have overlooked the potential inconsistency between individuals’ cognitive levels and behavioral patterns. This phenomenon could be an important factor affecting the practical effectiveness of current STDs prevention and intervention methods.

The presence of erroneous thought patterns and behaviors among MSM has become a key factor influencing their susceptibility to STDs (28, 29). In the real world, various positive, neutral, and negative situational events influence the cognitive interpretation process of MSM. The occurrence of risky behaviors may be driven by negative and erroneous beliefs. Implementing effective psychological intervention methods may have a positive impact on changing high-risk behaviors among MSM populations. However, there is a lack of systematic research on the cognition and behaviors of MSM, and even fewer professional personnel trained to conduct STDs prevention interventions, which is one of the bottlenecks in STDs prevention. Therefore, there is an urgent need to establish a structured, resource-efficient STDs prevention intervention approach to help MSM reduce the risk of STDs transmission.

A key conceptual premise of this study is that CBT-induced behavioral changes may exert measurable effects on the gut and perianal microbiota through several plausible pathways. First, reductions in unprotected anal intercourse and high-risk sexual practices directly alter the local mucosal environment—including changes in luminal pH, oxygen tension, and exposure to foreign microbial communities—which can reshape the ecological niche for resident microbiota. Second, behavioral modification may reduce the frequency of mucosal microtrauma and inflammatory responses, thereby lowering the availability of nutrients and adhesion sites for opportunistic pathogens. Third, CBT-related improvements in psychological distress and stigma coping may modulate the gut-brain axis via stress hormone pathways (e.g., cortisol, catecholamines), which are known to influence gut microbial composition and barrier function. Collectively, these pathways provide a biologically plausible framework linking cognitive-behavioral intervention to microbiota ecology. Therefore, we propose a conceptual model: CBT corrects maladaptive cognitive and decision-making processes, promotes behavioral improvement, which may in turn influence the gut/anorectal microbiota through the combined effects of local environmental changes, reduced inflammation, and neuroendocrine regulation. This model is explicitly exploratory and hypothesis-generating, and we acknowledge that the current cross-sectional post-intervention design cannot establish causality. Nevertheless, it offers a testable framework for future longitudinal studies that incorporate baseline microbiome sampling and mechanistic biomarkers.

In this study, we developed an intervention method for high-risk STDs behaviors among MSM populations based on CBT principles. By modifying the participants’ irrational cognitions or thought patterns, we aim to improve their unreasonable emotional and behavioral approaches, enhance their self-management and control abilities, reduce high-risk behaviors, and thereby prevent the spread of STDs. The results of this study investigated the occurrence of unprotected anal intercourse among participants in two groups during the intervention period from a behavioral dimension. The findings showed that the condom usage rate in the intervention group was significantly higher than that in the control group (*p* < 0.05), suggesting that the CBT-based intervention may have a role in reducing the occurrence of high-risk sexual behaviors.

Furthermore, studies have found that the unique sexual behavior patterns of MSM lead to significant changes in the composition of the gut microbiota by altering the anal/rectal environment (30, 31). Analyzing the gut microbiome of MSM provides a new perspective for the prevention of STDs. The research results of Kangjie Li′s team showed that the gut microbiome of HIV-negative MSM is dominated by Segatella (32). Zhihua Zhang’s team compared the differences in gut microbiota structure between the MSM group and the Non-MSM group, finding that the relative abundance of Actinobacteria, Proteobacteria, and genera such as Collinsella, Prevotella, Bifidobacterium, and Ralstonia was higher in the MSM group, while the relative abundance of Bacteroidetes and Bacteroidaceae was lower (33). In our study, we initially explored the impact of changes in MSM risky sexual behavior patterns under cognitive-behavioral therapy-based course interventions on the anal and rectal microbiome. The results showed that while the intervention group of MSM reduced risky behaviors, they had relatively higher gut microbiota richness. Based on previous literature, Bacteroides has been hypothesized to play dual roles in intestinal homeostasis. Moderate abundance is thought to support mucosal barrier function, whereas excessive proliferation of certain strains (e.g., *B. caccae*)—which has been associated with frequent unprotected anal intercourse in prior studies—may impair rectal mucus integrity and potentially trigger mucosal inflammation, thereby possibly elevating susceptibility to sexually transmitted pathogens. However, these mechanistic links are speculative in the context of our compositional data and require functional validation. The reduced abundance of *B. caccae* observed after CBT intervention might tentatively suggest concurrent changes in exposure patterns and mucosal status, but this interpretation remains speculative. *Coprococcus* is a key butyrate-producing beneficial commensal that maintains rectal mucosal integrity and suppresses inflammatory activation. Its abundance was markedly depleted in MSM engaging in frequent unprotected anal intercourse—an association that has been hypothesized in previous literature but remains exploratory in the context of our data. The reduction in high-risk sexual behaviors following the CBT intervention was accompanied by a higher abundance of *Coprococcus*. Whether this association reflects improved mucosal status remains a speculative hypothesis that requires direct mucosal biomarker measurements in future studies. The intergroup microbial taxonomic unit differences observed post-intervention can be considered exploratory, hypothesis-generating findings; however, they still require confirmation in longitudinal studies with baseline microbiome data. To sum up, these exploratory observations provide valuable clues for our future research direction.

Our study has the following limitations. First, this study aimed to explore whether gut microbiota characteristics might serve as potential exploratory indicators for monitoring behavioral changes, rather than to establish them as validated objective biomarkers. Although we observed significant differences in behavioral patterns and distinct characteristics of gut and perianal microbiota ecology among MSM under different intervention models, the results regarding the microbiota ecology lack baseline microbiota data and longitudinal comparative data. Besides, we did not collect or adjust for participants’ anal sex roles (receptive, insertive, or versatile) during data collection and statistical analyses. This discrepancy may arise from baseline variability or other confounding factors, making it impossible to determine the causal relationship between gut microbiota composition and changes in risky behavior patterns among MSM. Additionally, our study on the microbiome is limited by a small sample size, which increases the risk of false positives. Thirdly, the intervention program designed based on CBT in this study has characteristics of being structured, problem-oriented, and short-term, with the intervention process lasting only 4 weeks. This significantly limits the stability and interpretability of changes in participants’ microbiomes. The short-term changes in the microbiota ecology of the MSM population in the current study are only exploratorily associated with behavioral changes. In addition, Respondent-Driven Sampling (RDS) was employed solely as a recruitment strategy to access the hidden MSM population. It should be emphasized that RDS was not involved in subsequent group allocation, and therefore did not compromise inter-group comparability. However, this convenience-based sampling approach may have over-represented individuals with active social networks, which somewhat limits the generalizability of our findings to the broader MSM population, including those who are socially isolated or less networked. Thus, our results should be interpreted as preliminary and exploratory, and future multi-center studies using probability-based sampling are warranted to validate these findings. Finally, this study relied on self-reported questionnaires collected from participants during the short-term intervention period, which may introduce bias if participants’ responses are unreliable. This study examines changes in gut and perianal microbiota characteristics in relation to CBT-based behavioral interventions among MSM. It provides a valuable experience and model for future related research. We encourage future studies to establish larger sample sizes, implement more rigorous bias control, extend the duration of interventions and follow-ups, conduct paired longitudinal analyses before and after intervention, and thus more rigorously validate the causal association between CBT intervention and microbiome changes.

## 5 Conclusion

The CBT-based intervention developed by our team suggests potential as a strategy that may help curb high-risk sexual behaviors prevalent in MSM, although further confirmatory studies are needed. In addition, we conducted an initial exploratory analysis of the intergroup differences in the microbial characteristics of the anal and rectal regions of MSM as they change with high-risk sexual behaviors. This exploratory analysis offers a preliminary and exploratory perspective on potential biological correlates, rather than validated objective biomarkers, for evaluating behavioral intervention outcomes; we acknowledge that these correlational findings are subject to significant limitations in the current study. In this exploratory study, we attempted to integrate CBT-based methodology with metagenomic next-generation sequencing analysis techniques to establish a preliminary intervention framework for high-risk sexual behaviors in MSM. This effort provides a preliminary conceptual framework that may inform future research on long-term STD prevention and intervention strategies for MSM, but requires further validation in larger, long-term studies.

## Data Availability

The data that support the findings of this study are available on request from the corresponding author. The data are not publicly available due to privacy or ethical restrictions.

## Footnotes

1 http://www.randomization.com.

2 https://cloud.majorbio.com.

3 http://www.mothur.org/wiki/Calculators.
