# Supplementary material for: The effectiveness of the intervention model based on cognitive behavioral therapy in reducing risky sexual behaviors among MSM
Source: Front Public Health. 2026 Sep 3;14:1870879. doi: 10.3389/fpubh.2026.1870879 (PMC13581810; doi:10.3389/fpubh.2026.1870879)
Supplement: Supplementary file 1 [file Supplementary_file_1.docx]

Supplementary Material

# 1 Supplementary Figures and Tables

## 1.1 Supplementary Figures

**Supplementary Figure 1.** Pan/Core analysis of fecal samples and anal swab samples. Supplementary figure A-D：(A) Pan species curve of fecal samples; (B) Pan species curve of anal swab samples; (C) Core species curve of fecal samples; (D) Core species curve of anal swab samples.

**Supplementary Figure 2.** Graph of Alpha diversity rarefaction curves. Supplementary figure A-B：(A) Shannon curve. The abscissa represents the number of detected samples, the ordinate represents the Shannon index. (B) Simpson curve. Each curve represents a sample, and four different colors represent four groups respectively.

**Supplementary Figure 3.** Permutation Test of PLS-DA Analysis and Identification of Key Discriminatory Features. Supplementary figure A&C：Validation of the PLS-DA model by permutation testing. (A) Fecal sample. (C) Anus swab sample. Scatter plot illustrating the permutation test results used to evaluate the robustness and risk of overfitting of the PLS-DA model. The x-axis denotes the correlation (similarity) between the permuted and original class labels. The y-axis displays the goodness-of-fit (R²Y, grey diamonds) and predictability (Q², black diamonds) parameters. The rightmost points (similarity = 1.0) represent the metrics of the actual, unpermuted model. Supplementary figure B&D：Key discriminant features identified by PLS-DA. (A) Fecal sample. (C) Anus swab sample. The bar chart displays the top 20 operational taxonomic units (OTUs) ranked by their variable importance in projection (VIP) scores, derived from the partial least squares discriminant analysis (PLS-DA) model. The y-axis represents the VIP score, which quantifies the relative contribution of each feature to the group separation. OTUs with a VIP score > 1.0.

## 1.2 Supplementary Tables

Supplementary Table 1. Screening of Biomarkers in Fecal and Anal Swab Samples

| Species name | Group | LDA value | P value |
| --- | --- | --- | --- |
| *g__Coprococcus* | int_FB | 3.671 | 0.007 |
| *g__Desulfovibrio* | int_FB | 2.443 | 0.013 |
| *s__Desulfovibrio_piger* | int_FB | 2.493 | 0.013 |
| *s__Agathobaculum_butyriciproducens* | int_FB | 3.045 | 0.018 |
| *s__Coprococcus_comes* | int_FB | 3.372 | 0.019 |
| *g__Agathobaculum* | int_FB | 3.011 | 0.023 |
| *f__Bacteroidaceae* | con_FB | 4.457 | 0.028 |
| *g__Peptostreptococcus* | con_FB | 2.759 | 0.030 |
| *s__Peptostreptococcus_stomatis* | con_FB | 2.614 | 0.030 |
| *g__Erysipelatoclostridium* | con_FB | 2.847 | 0.031 |
| *f__Christensenellaceae* | int_FB | 2.397 | 0.031 |
| *g__Christensenella* | int_FB | 2.423 | 0.031 |
| *s__Coprococcus_catus* | int_FB | 2.723 | 0.032 |
| *g__Bacteroides* | con_FB | 4.093 | 0.041 |
| *s__Prevotella_copri* | int_FB | 5.130 | 0.042 |
| *f__Eggerthellaceae* | int_FB | 2.494 | 0.048 |
| *o__Eggerthellales* | int_FB | 2.522 | 0.048 |
| *g__Phascolarctobacterium* | int_FB | 3.245 | 0.049 |
| *g__Lachnoclostridium* | con_GS | 2.456 | 0.019 |
| *g__Levyella* | int_GS | 3.040 | 0.031 |
| *o__unclassified_p__Firmicutes* | con_GS | 2.404 | 0.031 |
| *f__unclassified_p__Firmicutes* | con_GS | 2.460 | 0.031 |
| *c__unclassified_p__Firmicutes* | con_GS | 2.436 | 0.031 |
| *g__Parvimonas* | int_GS | 2.218 | 0.036 |
| *s__Bifidobacterium_longum* | con_GS | 4.105 | 0.048 |
| *s__Peptostreptococcus_anaerobius* | int_GS | 2.877 | 0.015 |
| *s__Levyella_massiliensis* | int_GS | 3.275 | 0.031 |
| *s__Parvimonas_micra* | int_GS | 2.828 | 0.036 |
| *s__Bacteroides_caccae* | int_GS | 2.505 | 0.013 |

Supplementary Table 2. CBT Course Design

| Course Title | The purpose of the course | The content of the course |
| --- | --- | --- |
| 1. An explanation of the principles of CBT | The purpose is to enable participants to understand cognitive-behavioral theory and become familiar with the intervention process. | Cognitive Science and Behavioral Science, Overview of CBT, Principles and Steps of CBT (Core Beliefs, Intermediate Beliefs, Automatic Thoughts), Implementation Steps of CBT (Identifying Unfavorable Beliefs, Correcting Erroneous Beliefs, Developing New Cognitive Patterns, Improvement in Emotions and Behaviors, Enhanced Social Adaptability). |
| 2.Basic knowledge and prevention methods of AIDS/sexually transmitted diseases | Increase the level of knowledge among participants regarding AIDS/STDs prevention, help intervention recipients recognize the risks of high-risk sexual behaviors, and encourage them to change their risky behaviors. | AIDS concepts, infection mechanisms, sources of infection, transmission routes, current domestic and international infection status, prevention measures for the MSM population, AIDS testing and treatment; what are high-risk behaviors, low-risk behaviors, and safe behaviors; the risk level of various sexual intercourse methods in transmitting STDs/AIDS; the harm of high-risk behaviors to STIs/AIDS infection; how to avoid high-risk sexual behaviors. |
| 3. Other Basic Knowledge and Methods for Preventing STDs | Enhance participants' awareness of STDs; encourage research subjects to develop correct and healthy sexual perspectives. | Classification, etiology, clinical manifestations, diagnosis, treatment, and prevention of STDs; correct sexual morality and attitudes (health, equality, objectivity, responsibility). |
| 4. Conceptualizing the Case Problem | Utilize the problem conceptualization process to clarify the overall context of issues encountered by participants in their lives or studies; enhance participants' logical thinking and reasoning abilities in problem-solving; and provide participants with an initial understanding of the theoretical framework of cognitive-behavioral theory. | Explain the definition and principles of problem conceptualization to the participants; present some cases or scenarios related to AIDS and analyze them according to the problem conceptualization process. |
| 5. Identify Automatic Thoughts | Automatic thoughts are the thinking habits of most individuals, which are difficult to recognize and control before negative reactions occur. This course helps intervention group members identify spontaneously emerging automatic thoughts and discover and confirm those thoughts that hinder the cognitive and behavioral functioning of the thinking individual. | ① Identify emotions and reactions, helping intervention group members first recognize their emotions and reactions, as well as the underlying thoughts that trigger these emotions and reactions; ② Identify characteristics of irrational automatic thoughts, assisting intervention group members in recognizing three features of irrational automatic thoughts: absolute demands, overgeneralization, and catastrophic thinking. |
| 6. Identify Intermediate Beliefs | Intermediate beliefs lie between automatic thoughts and core beliefs. They are less noticeable than automatic thoughts but have a greater influence. Intermediate beliefs consist of rules, attitudes, and assumptions. This course aims to help intervention group members correctly identify and locate their erroneous intermediate beliefs when events and situations occur. | Identify the following cognitive errors: ① Black-and-white thinking, which involves viewing things in extreme, polarized terms without considering gradations or leaving room for middle ground; ② Catastrophic thinking, which uses negative thought to predict future outcomes while ignoring other possible results; ③ Labeling, which involves attaching definitive, generalized labels to oneself or others without subjectively considering positive possibilities; ④ Emotional reasoning, where the subject becomes overly sensitive to emotions related to a situation or event and thus accepts the cognitive implications of those emotions while ignoring or underestimating evidence from the opposite side; ⑤ Overgeneralization, where the subject blindly projects negative beliefs derived from an occasional incident onto other dissimilar situations; ⑥ Mind reading, where the subject draws conclusions without supportive evidence; ⑦ Magnification or minimization, where the subject's perception of an event or situation is excessively exaggerated or unduly reduced compared to its actual meaning. |
| 7. Identify and Correct Core Beliefs | Typically, members of the intervention group find it difficult to articulate their core beliefs, yet they hold these beliefs as absolutely true and correct within events or situations. This course aims to help intervention group members accurately locate and identify their core beliefs during events and situations, and learn how to correct maladaptive core beliefs. It also assists them in examining errors within their beliefs and constructing adaptive alternative beliefs. | Identification: ① Identify the central theme by noting recurring intrusive thoughts of intervention group members as potential clues to core beliefs in thinking records; ② The downward arrow technique, a peeling onion-like method of repeated questioning, to progressively delve into core beliefs. Correction: ① Reality testing, which involves using hypothetical reasoning where intervention group members test their existing beliefs in therapist-designed behavioral patterns or scenarios to recognize whether their original beliefs align with reality and make necessary changes; ② Positive self-talk, where on one hand, intervention group members consistently review and record their positive and optimistic aspects daily, and on the other hand, identify negative thoughts and propose alternative positive thoughts. |
| 8. System Desensitization and Behavioral Shaping Training | Assist intervention group members in gradually weakening and eliminating sensitive reactions in relatively opposing inhibitory situations, ultimately severing the link between the stimulus and the intervention group members' adverse reactions; help intervention group members form and establish a new behavioral habit. | By gradually reducing adverse reactions through stimuli of increasing intensity within the adaptive range of intervention group members, a hierarchy of sensitivity responses is first established, forming the basis and direction for systematic desensitization therapy. Subsequently, during situational exposure, relaxation training is progressively applied from lower to higher intensity stimuli, ultimately enabling the ability to relax both mind and body proficiently. To cultivate new positive behavioral habits, a method of breaking down tasks into manageable parts can be employed. First, clarify the overarching goal, then divide it into multiple smaller sub-goals, and develop specific implementation plans based on individual circumstances. Complete these steps sequentially from easy to difficult, rewarding oneself after each completed step to reinforce the belief, until the highest goal is ultimately achieved. |

**Supplementary Figure 3. Recruitment and Intervention Process**
