# Supplementary material for: The effectiveness of the intervention model based on cognitive behavioral therapy in reducing risky sexual behaviors among MSM
Source: Front Public Health. 2026 Sep 3;14:1870879. doi: 10.3389/fpubh.2026.1870879 (PMC13581810; doi:10.3389/fpubh.2026.1870879)
Supplement: Supplementary file 2 [file Supplementary_file_2.DOCX]

**Design and Determination of Intervention Effect Evaluation Tools**

**1. Regarding how to construct an initial entry pool**

(1) Literature Review Method. The research team screened through full-text journal databases such as Web of Science, PubMed, China National Knowledge Infrastructure (CNKI), VIP, and Wanfang. They retrieved literature using keywords or abstracts related to men who have sex with men (MSM), sexually transmitted infections (STIs), human immunodeficiency virus/acquired immunodeficiency syndrome (HIV/AIDS), intervention, preventive knowledge, and health literacy. The retrieval strategy involved using single subject terms, combined subject terms, and the combination of subject terms with abstracts to summarize relevant literature. Concurrently, based on the eight basic knowledge points for preventing AIDS in China, an initial item pool for the 'Health Literacy Scale for Sexually Transmitted Diseases and AIDS Prevention among MSM Population' was established.

(2) In-depth Individual Interview Method. The purpose is to understand the attitudes of MSM individuals toward high-risk sexual behaviors, prominent beliefs, and resistance to change factors, and to collect the current status, opinions, and suggestions of MSM individuals regarding the prevention of STIs/AIDS. We selected 3 to 5 MSM individuals with diverse characteristics and conducted in-depth interviews with them by trained research team members. The purpose of the interviews was to comprehensively understand various aspects of the participants' health education status, psychological and behavioral characteristics, community influences, and willingness to accept STIs/AIDS prevention and control services. We collected the belief composition of high-risk groups within the MSM population, with a focus on gathering beliefs from the interviewed population regarding three open-ended questions: attitudes toward high-risk behaviors for STIs/AIDS, methods that can raise awareness of STIs/AIDS prevention, and factors that promote or hinder the occurrence of high-risk behaviors. The information collected from interviews is used to revise and improve the initial item pool of the Scale.

**2. Regarding how to compile expert consultation questionnaires**

The questionnaire content includes: (1) Research Introduction: A brief summary of the research background, purpose, and content. (2) Instructions for Completing the Questionnaire. (3) Basic Information of Experts: Including gender, workplace, years of work experience, professional field, professional title, and highest academic degree. (4) The level of familiarity of experts with the research topic and their basis for judgment: The level of familiarity is categorized as very familiar, fairly familiar, generally familiar, somewhat unfamiliar, and unfamiliar; We converted the questionnaire content into a self-administered electronic questionnaire, which was distributed to the experts in the form of a QR code. Data collection, organization, and analysis were conducted using Excel tables and SPSS.

**3. Scoring of questionnaire item indicators**

The importance of each item in the Scale is evaluated using a 5-point Likert rating system, with the following scoring criteria: 5 points representing 'Very Important', 4 points representing 'Important', 3 points representing 'Generally Important', 2 points representing 'Not Important', and 1 point representing 'Very Not Important'. Based on this scoring system, we use the average importance score, selection rate, and full-score rate as evaluation criteria for the importance of each questionnaire item.

Average importance score = Total score / Total number of experts; Selection rate = Number of experts who assigned an importance score of 4 or higher / Total number of experts; Full-score rate = Number of experts who assigned an importance score of 5 / Total number of experts.

**4. Assignment of key statistical indicators related to experts**

The primary statistical indicators for expert selection are as follows:

(1) Expert Authority Coefficient (Cr): Derived from experts' self-evaluation, it is determined by three dimensions: the expert's academic attainment level (q1), judgment basis (q2), and familiarity with the research topic (q3). Referencing relevant literature, this study deems that an Expert Authority Coefficient ≥ 0.7 indicates that the expert's opinion is acceptable for adoption.

The individual authority coefficient of an expert = (q1 + q2 + q3) / 3

The group authority coefficient of experts = Sum of individual expert authority coefficients / Number of experts

(Note: q1 is the academic level weight, q2 is the judgment basis weight, and q3 is the familiarity weight)

**5. Selection criteria for each item of the questionnaire**

The selection criteria for each item of the questionnaire are as follows: (1) The average score for importance is ≥4 points; (2) The coefficient of variation is ≤0.25; (3) The selection rate is ≥80% and/or the full-score rate is ≥50%.

Items that meet all three criteria are directly included in the scale and do not proceed to the next round of expert consultation. Items that meet one or two of the above criteria are, after incorporating expert opinions and discussion by the research team, revised and then included in the next round of expert consultation. Items with an average importance score of less than 4 points are deleted and not included in subsequent rounds of expert consultation or the scale.

**6. Expert Profile**

A total of 8 experts from the fields of public health and preventive medicine, clinical medicine, and basic medicine were recruited to form the expert advisory panel. Their years of experience ranged from 12 to 30 years, with an average of (21.5 ± 5.29) years. The basic information on the experts is shown in the table below (Table 1.4):

Table 1.4: Expert Profile Information Form

| **Project** | **Number of people** |
| --- | --- |
| **Gender** |  |
| man | 3 |
| woman | 5 |
| **Work unit** |  |
| colleges and universities | 5 |
| hospital | 2 |
| medical Institutions | 1 |
| **Years of Experience** |  |
| 12～20 | 2 |
| 20～30 | 6 |
| **Professional Title** |  |
| senior | 5 |
| intermediate | 3 |
| **Highest Level of Education** |  |
| doctoral student | 4 |
| master's Student | 4 |

**7. Expert Positivity Coefficient**

This study uses the valid questionnaire response rate to indicate experts’ enthusiasm for participating in the project; the higher the valid questionnaire response rate, the greater the experts’ enthusiasm. A total of two rounds of expert consultation were conducted. In the first round, 10 questionnaires were distributed, and 8 were returned, resulting in a valid response rate of 80%; in the second round, 8 questionnaires were distributed, and all 8 were returned, resulting in a valid response rate of 100%. This demonstrates the experts’ high level of engagement and their strong support for and interest in the study.

**8. Level of Expertise**

Both rounds of expert consultation involved the same group of experts, and the authority coefficients were both 0.894, indicating that the experts who participated in the consultation had a high authority index, which meets the research criteria. Specifically, the experts’ academic proficiency ranges from 0.8 to 1, the values for the basis of their judgments range from 0.85 to 1, and their level of familiarity ranges from 0.6 to 1. (Table 1.5)

Table 1.5: Level of Authority Among Expert Groups

|  | q1 | q2 | q3 | Cr |
| --- | --- | --- | --- | --- |
| First Round | 0.925 | 0.956 | 0.80 | 0.894 |
| Second Round | 0.925 | 0.956 | 0.80 | 0.894 |

**9. Results of the First Round of Expert Consultations**

The first round of the expert questionnaire included a total of 27 items across three dimensions, comprising 16 knowledge items, 8 attitude items, and 2 behavior items.

(1) Knowledge Dimension: All 16 items were included in the scale. The importance scores for each item ranged from 4.125 to 4.875, the coefficient of variation (CV) ranged from 0 to 0.209, the selection rates ranged from 87.5% to 100%, and the perfect-score rates ranged from 50% to 87.5%.

(2) Attitude Dimension: Of the 8 items, 4 were included in the scale, 3 were deleted, 1 was revised and advanced to the next round of expert review, and 1 new item was added and advanced to the next round of expert review. The importance scores for the four items included in the scale ranged from 4.125 to 4.75, with coefficients of variation (CV) ranging from 0.091 to 0.159, selection rates ranging from 87.5% to 100%, and full-score rates ranging from 50% to 87.5%. The three items that were deleted were: “Do you need information about contraception?" “Have you ever been discriminated against because of your sexual orientation?” and “Have you considered coming out to others?” The importance scores for these items ranged from 3.125 to 3.75, which did not meet the criteria for inclusion in the second round of consultation; therefore, they were excluded. The one item requiring modification is “Do you need information on STD/HIV prevention?" which has an importance score of 4.125, a coefficient of variation of 0.240, a selection rate of 87.5%, and a perfect score rate of 37.5%. Based on expert recommendations, one new item has been added: “Do you believe that contraceptive measures are the same as HIV prevention measures?”

(3) Behavioral Dimension: Of the two items, one was included in the 'Scale,' one was deleted, and one new item was added, and we will proceed to the next round of expert consultation. The importance score for items included in the ‘Scale’ was 4.375, with a coefficient of variation of 0.170, a selection rate of 87.5%, and a perfect score rate of 50%. The item that was deleted was "Age at first sexual intercourse?" which had an importance score of 3.875. Based on expert recommendations, a new question has been added: “How often have you used condoms during sexual intercourse in the past month?”

**10.Results of the Second Round of Expert Consultations**

The second round of the expert questionnaire included a total of 3 items across two dimensions, comprising 2 attitude items and 1 behavioral item. One item was removed, and two items were added; the specific items have been finalized, and the expert consultation process has concluded. Following two rounds of expert consultation, the final 'scale' comprising 23 items across three dimensions was developed.

(1) Attitude Dimension: Following discussions based on expert opinions, the research team revised the item to read, “Do you need to proactively learn about the prevention of sexually transmitted diseases and HIV/AIDS?” It was assigned an importance score of 4.5, with a coefficient of variation of 0.119, a selection rate of 100%, and a perfect score rate of 50%, and was included in the 'Scale.' The newly proposed item was assigned an importance score of 3.125 and was therefore excluded.

(2) Behavioral Dimension: The importance score for the newly added item was 4.375, with a coefficient of variation of 0.170, a selection rate of 87.5%, and a perfect score rate of 50%; it was included in the 'Scale.'

The final version of the Scale consists of four domains: basic demographic information, knowledge of STDs/HIV, attitudes toward STDs/HIV, and STD/HIV-related behaviors, with a total of 30 items. (1) The basic demographic module contains seven items covering participants’ age, living arrangements, income level, relationship status, educational attainment, occupation, and history of same-sex sexual activity. (2) The STD and HIV/AIDS knowledge dimension examines content including the clinical stages of HIV/AIDS, transmission routes of STDs and HIV/AIDS, treatment options, and testing approaches. Each question is scored 1 point. Items 1–8 correspond to the Eight National Guidelines, while Items 9–16 assess advanced STD/HIV-related knowledge, amounting to 16 items in total. (3) The Attitudes domain mainly evaluates participants’ perceptions of sexual risks, willingness to use condoms, motivation to learn prevention and treatment knowledge, self-reported sexual orientation, and other relevant indicators, comprising five items. (4) The behavior domain primarily investigates whether participants engaged in high-risk sexual behaviors in the recent past and during the intervention period, and includes two items.
